# Identifying Candidates for Immunotherapy among Patients with Non-Melanoma Skin Cancer: A Review of the Potential Predictors of Response

**DOI:** 10.3390/jcm11123364

**Published:** 2022-06-11

**Authors:** Enrico Zelin, Carlo Alberto Maronese, Arianna Dri, Ludovica Toffoli, Nicola Di Meo, Gianluca Nazzaro, Iris Zalaudek

**Affiliations:** 1 Dermatology Clinic, Maggiore Hospital, University of Trieste, 34125 Trieste, Italy; enrico.zelin@gmail.com (E.Z.); ludovica.toffoli@gmail.com (L.T.); nickdimeo@libero.it (N.D.M.); iris.zalaudek@gmail.com (I.Z.); 2 Dermatology Unit, Fondazione IRCCS Cà Granda Ospedale Maggiore Policlinico, 20122 Milan, Italy; carlo.maronese@unimi.it; 3 Department of Pathophysiology and Transplantation, Università degli Studi di Milano, 20122 Milan, Italy; 4 Department of Medicine (DAME), University of Udine, 33100 Udine, Italy; arianna.dri@gmail.com; 5 Department of Medical Oncology, Azienda Sanitaria Friuli Centrale (ASUFC), 33100 Udine, Italy

**Keywords:** non-melanoma skin cancer, immunotherapy, basal cell carcinoma, cutaneous squamous cell carcinoma, Merkel cell carcinoma, predictors, biomarkers

## Abstract

Background: Non-melanoma skin cancer (NMSC) stands as an umbrella term for common cutaneous malignancies, including basal cell carcinoma (BCC) and cutaneous squamous cell carcinoma (cSCC), together with rarer cutaneous cancers, such as Merkel cell carcinoma (MCC) and other forms of adnexal cancers. The majority of NMSCs can be successfully treated with surgery or radiotherapy, but advanced and metastatic stages may require systemic approaches such as immunotherapy with immune checkpoint inhibitors (ICIs). Summary: Since immunotherapy is not effective in all patients and can potentially lead to severe adverse effects, an important clinical question is how to properly identify those who could be suitable candidates for this therapeutic choice. In this paper, we review the potential features and biomarkers used to predict the outcome of ICIs therapy for NMSCs. Moreover, we analyze the role of immunotherapy in special populations, such as the elderly, immunocompromised patients, organ transplant recipients, and subjects suffering from autoimmune conditions. Key messages: Many clinical, serum, histopathological, and genetic features have been investigated as potential predictors of response in NMSCs treated with ICIs. Although this field of research is very promising, definitive, cost-effective, and reproducible biomarkers are still lacking and further efforts are needed to validate the suggested predictors in larger cohorts.

## 1. Introduction

Non-melanoma skin cancers (NMSCs) are the most common human malignancies, accounting for up to 30% of all tumors [1,2]. Basal cell carcinomas (BCCs) and cutaneous squamous cell carcinomas (cSCCs) represent the vast majority of NMSCs. On the other hand, Merkel cell carcinomas (MCCs) are rarer tumors belonging to the category, but given their aggressive behavior, local recurrences, lymph nodal involvement, and distant metastases are very frequent during the course of the disease [1,2,3,4,5].

The incidence of BCCs and cSCCs, but also of MCCs, is steadily growing, which is probably due to many factors, such as the progressively aging population, the cumulative ultraviolet (UV) radiation exposure, and the increasing use of immunosuppressive therapies; moreover, earlier diagnosis may also play a role [1,2,6,7]. Although the majority of NMSCs can be effectively treated with surgery or radiotherapy, advanced and metastatic tumors require systemic treatments, and immune checkpoint inhibitors (ICIs) represent an actual choice.

In this paper, we aim to summarize existing evidence regarding the identification and selection of patients that could benefit from ICIs therapy. This topic is an emerging field, and we are likely to see new developments in the near future, which will hopefully be to the benefit of the patients. For this review, a search of PubMed, Science.gov, and ClinicalTrials.gov databases was performed using the terms ‘immune checkpoint inhibitors’, ‘immunotherapy’, ‘anti-PD-1′, ‘anti-PDL-1′ in combination with ‘non-melanoma skin cancer’, ‘basal cell carcinoma’, ‘cutaneous squamous carcinoma’, or ‘Merkel cell carcinoma’. Only articles in English were selected. Other potentially relevant articles were identified by manually checking the references of the included literature.

## 2. The Role of Immunotherapy in Non-Melanoma Skin Cancer

Several lines of evidence suggest that most skin cancers such as cSCC, BCC, and MCC have a high mutational burden, which in turn has been shown to predict the response to ICIs [8,9,10]. Further support of a link between immune response and NMSC development is provided by data showing that immunosuppression is an important risk factor for these malignancies. For example, cSCC and MCC incidence increases by tens to hundreds of times in individuals with T-cell dysfunction, such as solid organ transplant recipients, HIV positive patients, subjects with chronic lymphocytic leukemia, or other hematologic malignancies [3,4,5,11].

Immunotherapy has become a cornerstone for the treatment of many cancers, including cutaneous malignancies, and is currently indicated for melanoma, cSCC, BCC, and MCC treatment [12,13,14,15,16]. Immunotherapy drugs act by inhibiting immune checkpoints, eventually improving the activity of the immune system against the tumoral cells and reducing regulatory-T-cell-mediated immunosuppression [13]. Specific targets are cytotoxic T-lymphocyte–associated protein 4 (CTLA-4) for ipilimumab, programmed cell death receptor-1 (PD-1) for cemiplimab, nivolumab and pembrolizumab, and programmed cell death ligand-1 (PD-L1) for avelumab [13]. CTLA-4 is a T-cell protein receptor that prevents T-cell activation when bound to a co-stimulatory protein receptor called B7, which is localized on the surface of an antigen-presenting cell. The PD-1/PD-L1 pathway is similarly involved in immune tolerance. PD-L1 is expressed on antigen-presenting cells and neoplastic and tumor-infiltrating immune cells; on the other hand, PD-1 is expressed on CD8+ and CD4+ T-cells, B-cells, and natural killer (NK) cells [17,18,19,20,21].

According to recent guidelines from the European Association of Dermato-Oncology, patients with locally advanced cSCC (la-cSCC, not amenable to curative surgery or radiation therapy) and metastatic cSCC (m-cSCC) should receive first-line treatment with anti-PD-1 immunotherapy (grade of recommendation: A; level of evidence: 2) [22]. On the other hand, different systemic therapeutic regimens, such as Epidermal Growth Factor Receptor (EGFR) inhibitors and platinum-based chemotherapy have been implied, but in comparison, they appear less effective [22]. Currently, cemiplimab is the only anti-PD-1 agent approved for la-cSCC and m-cSCC both in USA and Europe and its use is also being evaluated in adjuvant and neo-adjuvant settings [23].

Regarding locally advanced and metastatic BCC (la-BCC and m-BCC), the mainstay of systemic therapy is represented by Sonic Hedgehog inhibitors (HHIs), but there is growing evidence of a potential role of anti-PD-1 regimens, in particular in HHIs-resistant tumors or patients who do not tolerate treatment due to adverse events [12]. In February 2021, the FDA has granted the approval for cemiplimab use in patients with la-BCC and m-BCC who were previously treated with HHIs or for whom HHIs are not appropriate in light of the positive results of a recent study (NCT03132636) [12].

Finally, as far as concerns MCC, immunotherapy is used for locally advanced (la-MCC) or metastatic disease (m-MCC) as well. Among several ICIs, avelumab is currently authorized both in Europe and in the USA, while pembrolizumab has only been approved by the FDA (Food and Drugs Administration) [4,5]. It appears that ICIs allow for durable responses with a more favorable toxicity profile than traditional chemotherapies, such as platinum-based regimens with the addition of etoposide [4,5]. Although MCC is a chemo-sensitive neoplasm, the progression-free survival and overall survival only reach a few months; therefore, chemotherapy is considered as second-line in MCC nowadays [3,4,5,24]. Regarding adjuvant and neo-adjuvant settings, ICIs therapies for MCC are still being investigated in ongoing trials [4,5].

In this article, we will examine the main ICIs and their use in NMSC treatment. In evaluating tumor response to treatment, the RECIST v.1.1 (Response Evaluation Criteria In Solid Tumors) criteria or other variants are often used, thereby defining the complete response (CR), partial response (PR), stable disease (SD), progressive disease (PD), response rate (RR = CR + PR), and disease control rate (DCR = CR + PR + SD) [25,26].

### 2.1. Ipilimumab

Ipilimumab is an anti-CTLA4 monoclonal antibody (IgG1k) approved for melanoma and renal cell cancers (in monotherapy or in association with nivolumab). Currently, the recommended dosing for metastatic or unresectable melanoma is 3 mg/kg intravenously (IV) every 3 weeks for a total of 4 doses [27].

Data regarding its use in NMSC are limited to case reports [28,29,30,31]. Patients with metastatic melanoma and concurrent advanced or metastatic cSCC or BCC had a durable remission of these malignancies when exposed to ipilimumab [28,29]. An ongoing study (NCT03521830) is currently evaluating ipilimumab in association with nivolumab in one of the arms for la-BCC or m-BCC. Although conflicting results have been reported in other neoplasms [32], dual PD-1 and CTLA-4 blockade with ipilimumab and nivolumab has gained some interest in head and neck SCC [33] and is currently being studied in cSCC (NCT04620200).

As combination approaches inherently imply a greater risk for immune-related adverse events (irAE), a combination of ipilimumab with probody^®^ therapeutics (i.e., antibody prodrugs designed to be activated by tumor-associated proteases) will possibly have a role in the near future in maximizing responses while keeping irAE to an acceptable level in cSCC and NMSC [34]. In MCC, ipilimumab has been seldom studied, with inconclusive results about its antitumor activity [30,31]. In addition, as adjuvant therapy for resected MCC, ipilimumab did not demonstrate activity [35]. Recently, combination immunotherapy with ipilimumab plus nivolumab has led to an overall response rate of 60% in a 5-patient series of avelumab refractory MCC cases, with 2 PR and 1 CR [36]. Moreover, cases of durable remission achieved with this approach possibly advocate for further studies into sequential, combined immunotherapy [37].

In summary, based on the current evidence, ipilimumab in monotherapy appears less promising than anti-PD1 drugs for NMSC (while combination regimens with other ICIs could be useful). Data regarding ipilimumab use are summarized in Table 1.

### 2.2. Cemiplimab

Cemiplimab is a human monoclonal anti-PD-1 antibody and is the only immunotherapy drug approved for treating la-cSCC (patients not eligible for curative surgery or radiotherapy) and m-cSCC at a dose of 3 mg/kg IV every 2 weeks. In the phase I/II trial EMPOWER-CSCC-1 (NCT02383212, NCT02760498) that led to drug approval, RR was 50% (95% confidence interval [CI]: 30–70%) and DCR was 65% (95% CI: 44–83%) for the phase I cohort (26 patients with la-cSCC and m-cSCC) [38,39]. For the phase II cohort (59 patients with m-cSCC), RR was 47% (95% CI: 34–61%) and DCR was 61% (95% CI: 47–74%) [38,40]. These data were confirmed by the analysis of the phase II la-cSCC EMPOWER-CSCC-1 cohort (78 patients) [41] and by a retrospective study involving 26 la-cSCC and m-cSCC treated with anti-PD-1 drugs [42]. Real-world data from a French study on 18 patients, including older and immunocompromised patients, are in line with these results, showing an even higher overall RR (67%) with a DCR of 72% (33% CR, 33% PR, 6% SD) [43]. Data from a larger real-life study from the French CAREPI group, including 245 patients with la-cSCC or m-cSCC, showed a DCR of 59% (21% CR, 29% PR, 9% SD) [44]. The results from a large Italian retrospective study involving 131 patients with unresectable cSCCs were similar, with an overall RR of 58% [45].

According to these promising results, cemiplimab is currently investigated in a neoadjuvant setting—currently, several ongoing trials are investigating this hypothesis (NCT04428671, NCT03565783) [46]. Interestingly, one of them adopts intralesional cemiplimab administration (NCT03889912). Cemiplimab has been proposed in an adjuvant setting as well (NCT03969004, NCT04428671, NCT04632433) [23].

Regarding BCC, cemiplimab was first administered in a patient with m-BCC that was resistant to therapy with Sonic Hedgehog inhibitors (HHIs). The patient achieved a partial response (PR) with 10 mg/kg administered every 2 weeks for 24 weeks [47]. The drug is also being used at a dose of 350 mg IV every 3 weeks in an ongoing phase II study involving la-BCC and m-BCC resistant to therapy with HHIs (NCT03132636). Data have been published for patients with la-BCC (84 subjects), highlighting an objective RR of 31% (95% CI: 21–42%), DCR of 79.8%, and median estimated progression-free survival (PFS of 19.3 months) for all patients; these results granted FDA approval as previously mentioned [12]. Moreover, cases of durable remission are increasingly being documented [48,49].

Finally, as far as MCC is concerned, cemiplimab has been investigated in only two patients, with PR and SD [50].

Table 2 summarizes the most significant literature regarding cemiplimab use in locally advanced and metastatic NMSC patients.

### 2.3. Pembrolizumab

Pembrolizumab is a humanized monoclonal anti-PD-1 antibody approved for advanced melanoma and other malignancies, including metastatic non-small cell lung cancer, Hodgkin’s lymphoma, renal cancer, gastric cancer, head and neck SCC, and MCC [52]; it is typically administered at a dosage of 2 mg/kg IV every 3 weeks.

Regarding cSCC, there are reports of patients treated with pembrolizumab achieving CR [53,54,55] and PR [52,53,56,57,58,59,60,61] or showing DP [53]. These outcomes led to the design of several studies. In the open-label, single-arm, phase II study, KEYNOTE-629, pembrolizumab was administered in 105 patients with recurrent or metastatic cSCC and 54 with la-cSCC. In the la-cSCC cohort, the objective RR was 50.0% (95% CI, 36.1% to 63.9%), with 16.7% of patients achieving a CR and 33.3% a PR. In the recurrent or metastatic cSCC cohort, the objective RR was 35.2% (95% CI, 26.2% to 45.2%), including 10.5% of patients with CR and 24.8% with PR [62,63]. Moreover, the phase II CARSKIN trial (NCT02883556), involving 57 patients with unresectable cSCC, showed comparable results, with an objective RR of 42% (95% CI: 29–56%) and DCR of 60% (95% CI: 46–72%) [64,65]. Interestingly, according to a prespecified subgroup analysis of the NCT02721732 open-label, phase II clinical trial, those patients with advanced cSCC that achieved CR (3/19) remained free of recurrence after a long-term follow-up, potentially experiencing cure [66]. Currently, ongoing studies are evaluating not only pembrolizumab monotherapy (NCT02964559), but also novel therapeutic approaches for la-cSCC, such as pembrolizumab associated with cetuximab [67], a human-murine chimeric monoclonal IgG1 antibody directed against EGFR (NCT03082534), with promising results reported for recurrent or metastatic head and neck SCC [68].

Pembrolizumab is also being employed in an adjuvant setting for la-cSCC after surgery or radiotherapy (NCT03833167).

Regarding BCC, there are reports of variable responses in patients treated with pembrolizumab for concurrent melanoma [69,70]. In la-BCC and m-BCC, pembrolizumab use has been initially described in individual cases or case series, with variable results [71] and reports of patients obtaining CR [72], PR [73,74], and PD [59]. In a non-randomized open label study involving 16 patients with la-BCC (NCT02690948), either pembrolizumab monotherapy or pembrolizumab plus vismodegib (Sonic Hedgehog inhibitor, HHI) were administered. For the first group, RR at 18 weeks was 44% (4/9 patients; 95% CI: 14–79%), while for the combination therapy group, it was 29% (2/7 patients; 95% CI: 4–71%) [75]. Moreover, an ongoing study is investigating pembrolizumab use for la-BCC in a neoadjuvant and adjuvant setting (NCT04323202) [46].

As far as la-MCC is concerned, several case reports demonstrated variable results for pembrolizumab (3 CR, 2 PR, 3 PD) [76,77,78,79,80,81,82]. In a multicenter phase II study (KEYNOTE 017) involving 50 patients with advanced MCC, CR was 30%, PR was 28%, SD was 8%, and PD was 32%. Objective RR was 58%, median PFS was 16.8 months, and the 3-year OS was 59.4% for all patients and 89.5% for responders [18,83,84]. In light of the efficacy showed, the United States Food and Drug Administration (FDA) approved pembrolizumab as a first-line therapy for advanced MCC in 2018. This anti-PD-1 drug is also being studied for resected MCC in an adjuvant setting (NCT03712605).

Table 3 summarizes the most significant literature regarding pembrolizumab use in locally advanced and metastatic NMSC patients.

### 2.4. Nivolumab

Nivolumab is a fully human monoclonal anti-PD-1 IgG4 antibody approved for advanced melanoma and other malignancies, including non-small cell lung cancer, Hodgkin’s lymphoma, renal cancer, head and neck SCC, urothelial carcinoma, colorectal cancer, and hepatocellular carcinoma. Depending on the cancer type, nivolumab is given IV at a dosage of 240 mg or 3 mg/kg every 2 weeks.

Concerning la-SCC treated with nivolumab in monotherapy or in combination with other drugs, there are reports [53,56,85,86,87] and also, ongoing studies showing the potential benefits (NCT03834233, NCT04204837, NCT02978625). This drug is also being investigated in the neoadjuvant setting for malignancy (NCT04620200).

As far as concerns la-BCC and m-BCC, nivolumab use has been described in some cases, with a certain degree of response [56,88]. Currently, ongoing trials are investigating its efficacy for la-BCC and m-BCC administered in monotherapy or with ipilimumab (NCT03521830, NCT03816332, NCT02834013) [27]. Interestingly, another currently active trial investigates a combination of ablative fractioned laser plus topical nivolumab for BCCs (NCT04570683).

In regard to MCC, nivolumab was administered in three patients with metastatic disease, allowing them to achieve PR [89,90,91]. Moreover, in the phase I/II CheckMate 358 study, nivolumab was used in 25 patients with advanced MCC, obtaining 14% CR, 55% PR, 18% SD, and 14% DP [92]. In the same Checkmate 358 study there was also a neoadjuvant arm: 47.2% (17/36) of the patients who underwent subsequent surgery achieved a complete pathologic response [93]. There are other currently ongoing trials evaluating nivolumab for MCC, which are also associated with other therapeutic approaches (among them, NCT03071406 and NCT02978625). In some studies, this anti-PD-1 drug is being employed for MCC in an adjuvant setting (NCT02196961) and also in combination with radiotherapy and ipilimumab, as previously stated (NCT03798639).

In a recent study (NCT03816332) involving eight kidney transplant recipients with advanced cutaneous cancers (melanoma, cSCC, MCC), nivolumab was used in combination with ipilimumab, thereby maintaining contemporary immunosuppression with tacrolimus and prednisone in order to prevent allograft loss, but the results were not encouraging [94].

Table 4 summarizes the most significant literature regarding nivolumab use in NMSC patients.

### 2.5. Avelumab

Avelumab is an anti-PD-L1 antibody that interferes with the PD-1 pathway, which is similar to pembrolizumab and nivolumab. It is currently approved for m-MCC, locally advanced or metastatic urothelial carcinoma, and, in association with axitinib, advanced renal carcinoma. It is administered IV at the dosage of 800 mg or 10 mg/kg every 2 weeks.

Individual case reports initially described one CR case [96] and one PR case [97] for m-MCC treated with avelumab. More data were collected in the JAVELIN Merkel 200 trial, a phase II prospective, open-label, multicenter study (NCT02155647) [20,98], which was conducted on 88 subjects with previously treated m-MCC (part A) and 116 subjects with naïve m-MCC (part B) [99,100]. The outcomes for the part A cohort were 11% CR, 22% PR, 10% SD, 36% PD, and a median OS of 12.9 months [20,98,99]. The outcomes for the part B cohort were 16% CR, 23% PR, 10% SD, 41% PD, and a median OS of 20.3 months [100]. Therefore, in 2017, on the basis of these new insights, avelumab received accelerated FDA approval for m-MCC; later, the drug was also approved in Europe with the same indication [99]. Meanwhile, the global avelumab program expanded access, which was designed to provide the compassionate use of avelumab prior to approval and led to similar response rates. The main treatment-related adverse events reported were infusion-related reactions and pyrexia [101,102].

Long-term data from the JAVELIN Merkel 200 trial (part A cohort) showed a median OS of 12.6 months (95% CI 7.5–17.1 months) and a 5-year OS rate of 26% (95% CI 17% to 36%). However, considerable differences in patients with PD-L1+ versus PD-L1- tumors were documented, with a median OS of 12.9 months (95% CI 8.7–29.6 months) and a 5-year OS rate of 28% (95% CI 17% to 40%) in the former versus a median OS of 7.3 months (95% CI 3.4–14.0 months) with a 5-year OS rate of 19% (95% CI 5% to 40%) in the latter [103,104].

The results from SPEAR-Merkel, the first study to evaluate real-world clinical outcomes in 94 patients with la-MCC and m-MCC receiving first-line avelumab (*n* = 28), non-avelumab immunotherapies (*n* = 26, 19 pembrolizumab, 7 nivolumab), or chemotherapy (*n* = 40), also highlight the therapeutic value of avelumab with an overall response rate of 64.3% and a median PFS of 11.4 months, compared to 61.5% with a median PFS of 8.1 months in the non-avelumab immunotherapy group and 42.5% with a median PFS of 6.1 months in patients undergoing chemotherapy [105]. In a similar real-world setting study involving a cohort of 20 patients with advanced MCC, comparable results were reported (overall RR: 65%; overall median time to treatment failure: 22 months) [106].

Of note, ongoing studies are evaluating avelumab in combination with other therapies for MCC (among them, trials NCT02584829, NCT04393753, NCT04261855, and NCT03853317). The abovementioned data are summarized in Table 5.

In a recent study, avelumab has also been used for MCC as a neoadjuvant treatment. In the 10 evaluated patients that underwent surgery, 4 (40%) reached a pathological CR [107]. Moreover, ongoing randomized phase II and III trials are currently evaluating its efficacy in the adjuvant setting (NCT03271372, NCT04291885).

Regarding other NMSCs, avelumab use is currently being investigated for la-cSCC and m-cSCC in phase II studies, either in monotherapy or in combination with other treatments, such as the EGFR inhibitor cetuximab (NCT03944941), which could speculatively synergize with the immunostimulatory effects of avelumab [108] and radiotherapy (NCT03737721).

### 2.6. Other Immunotherapies

New immunotherapy molecules are currently under investigation and they could have a potential role in NMSCs treatment in the near future. Among them, we can find other drugs directed against the PD-1/PD-L1 axis, such as anti-PD-1 monoclonal antibody INCMGA0001 (NCT03599713) and CK301—also called cosibelimab (NCT03212404) [30].

Besides the anti-CTLA4 and anti-PD-1/PDL-1 axis, several other inhibitory molecules are expressed by T-cells in the tumor micro-environment, such as lymphocyte activation gene-3 (LAG-3), T-cell immunoglobulin and mucin domain (TIM-3), or T-cell immunoreceptor with immunoglobulin and immunoreceptor tyrosine-based inhibition motif domains (TIGIT). According to emerging strategies, inhibitors targeting these different checkpoints could be used alone or in combination with anti-PD-1/PD-L1 drugs [109,110,111].

LAG-3 appears to be upregulated in activated natural killer (NK) cells, as well as activated CD4+ and CD8+ T-cells. In tumoral diseases and chronic infections, LAG-3 seems to plays a synergistic role with PD-1 to hinder immune response, therefore its inhibition could be useful as a therapeutic option [111]. Anti-LAG-3 molecules are being investigated for several advanced malignancies, including MCC (trials NCT03538028 and NCT02488759).

TIM-3 plays a role in T-cell exhaustion and its inhibition re-establishes T-cell cytotoxic effects; TIM-3 inhibitor efficacy is being assessed in the NCT03652077 trial for advanced malignancies, including MCC [109].

TIGIT plays a role in the activation and maturation of NK cells and T-cells. TIGIT and PD-1 are co-expressed in regulatory T-cells in various cancers; anti-TIGIT antibodies demonstrated synergistic effects with anti-PD-1/PD-L1 antibodies [108]. Currently, a phase I study is evaluating a monoclonal anti-TIGIT antibody (AB154) in monotherapy and in combination with an anti-PD-1 antibody (AB122) in patients with advanced malignancies, including MCC (NCT03628677).

Besides ICIs, novel immune-based strategies are currently emerging with different aims, such as the upregulation of antigen presentation and release by tumors (for example Toll-like Receptors [TLR] agonists), upregulation of pro-inflammatory signaling and cytokines (for example, interferons and interleukins), co-stimulation of T-cells (for example, agonistic agents targeting OX40, CD27, or glucocorticoid-induced tumor necrosis factor receptor [GITR]), reversal of other immunosuppressive signals expressed by tumoral cells themselves (for example, agents targeting adenosine receptors and the CD47-SIRPα pathway), and immune cell therapy (including adaptive T-cell therapy and innate immune cell therapy) [30].

Hopefully, we will see many new therapeutic options in the field of cancer immunotherapy over the course of the next few years, which will also lead to new perspectives for NMSCs.

## 3. General Considerations on Predictors and Biomarkers for Immune Checkpoint Inhibitors Efficacy in Solid Tumors

One of the most critical issues since the introduction of immunotherapy is identifying the ideal candidates that could benefit from the treatment. First of all, it is necessary to remember that immunotherapy can potentially lead to severe, irreversible, and sometimes fatal adverse effects; therefore, a careful risk/benefit evaluation is necessary. Indeed, ICIs are generally used when the burden of disease is high in metastatic or locally advanced settings, while their role as adjuvant or neoadjuvant regimens is currently not approved for NMSCs. In general, the two principles that should guide patient selection are benefit maximization and adverse effects reduction, namely finding biomarkers or other parameters that can predict therapeutic efficacy or, conversely, the probability of therapy failure or adverse effects.

There are scant data regarding the predictors of response for NMSCs, while research has produced more evidence for melanoma and other cancers for which immunotherapy is approved, such as non-small lung cancer and renal cancer. In this section, we will briefly discuss general predictive factors derived from other neoplastic settings that could be potentially implemented to NMSCs and subdivided into different categories—starting from widely accessible to more specific ones (Table 6).

### 3.1. Clinical Predictors and Special Populations

There are not many clinical predictors to define immunotherapy responses.

It is well known that ICIs can produce autoimmune-like toxicities, which are defined as immune-related adverse events (irAEs). These toxicities differ depending on the agent, malignancy, and individual susceptibilities, and any organ may be affected, although cutaneous and intestinal manifestations are the most common. Corticosteroids can be used for the treatment of moderate and severe irAEs, but sometimes the occurrence of these adverse events can lead to the discontinuation of immunotherapy [112].

Some studies linked the development of cutaneous adverse events to a better response to ICIs in many solid tumors [113], and low-grade irAEs were associated with a higher overall RR, indicating a responsive immune asset [114,115]. Nonetheless, these findings need a more robust validation; moreover, adverse effects arise only after treatment commencement, thereby reducing the usefulness of irAEs as a predictor.

Among the other clinical predictors, obesity was associated with an increased efficacy of anti-PD-1/PD-L1 treatment in both tumor-bearing mice and human patients. Such an obesity paradox is related to the male gender and higher serum creatinine, and has been observed in melanoma as well as in a variety of other neoplasms [116]. Obesity acts by impairing antigen-specific T-cell priming through PD-1/PD-L1 axis upregulation. Thus, PD-1 blockade in this setting results in both greater responsiveness and greater incidence of adverse reactions [117]. Interestingly, a real-life, multicenter, retrospective observational study involving more than one thousand patients has indeed revealed that higher body mass index was significantly related to the higher occurrence of any grade irAEs, grade 3 and grade 4 irAEs, and irAEs leading to treatment discontinuation [118]. Real-world data also showed that grade 3 irAEs were more common in ICIs responders [119].

Thanks to the diffusion of ICIs as a therapeutic approach for solid tumors and NMSC, a wider population is being treated and some evidence is developing regarding ICIs use in special groups that are traditionally excluded from these systemic treatments. Analyzing these new data could lead to new insights in patient selection. Among these special groups, we will briefly discuss elderly, immunocompromised individuals, organ transplant recipients, and patients suffering from autoimmune conditions. Of note, the evidence in these populations was generally collected analyzing solid tumors that are different from NMSC (mostly melanoma).

#### 3.1.1. Elderly

As far as age is concerned, it does not seem to play a role in the immunotherapeutic response, and in some cases, elderly patients can even show a better response to ICIs than younger ones. However, these data are derived from analyzing solid tumors that are different from NMSC. According to a retrospective analysis of patients with melanoma and lung cancer treated with anti-PD-1, immunotherapy in older and younger patients shows the same efficacy [120,121]. A small real-world study on 18 cemiplimab-treated patients with la-cSCC or m-cSCC even demonstrated an association between response and age above 60 years [43].

#### 3.1.2. Immunocompromised Patients

Immunotherapy for neoplastic diseases is traditionally avoided in immunocompromised patients due to concerns regarding reduced efficacy and safety (additional immune dysregulation in those patients who are already at higher risk for autoimmunity) [122]. However, there is emerging evidence in favor of the abovementioned therapeutic approach in this special population, although data are scarce for NMSCs [122,123].

According to a recent systematic review, ICIs are an effective treatment option in HIV-positive patients with advanced-stage cancer (including cSCC) and several ongoing prospective trials will shed further light on this topic [124].

As far as patients are concerned in terms of chronic immunosuppressive therapy, there is scarce evidence, which was derived from retrospective studies conducted on lung cancer. It is reported that early or baseline use of corticosteroids has an adverse impact on ICIs response rates and outcomes; moreover, patients who are receiving steroids have typically more aggressive diseases, representing a possible confounding effect when interpreting these data [125,126,127]. In these cases, it seems reasonable to switch to alternative medications or decrease the corticosteroid dose, if feasible [125]. Conversely, the late use of steroids during the treatment course is in contrast to improved outcomes. However, it has to be considered that steroids are often employed to treat irAEs, which are themselves positively associated with ICIs efficacy [127,128].

Regarding advanced cSCC, a real-world retrospective study involving 131 patients treated with cemiplimab also identified chronic corticosteroids therapy as a negative predictor that is significantly associated with a worse response [45].

#### 3.1.3. Organ Transplant Recipients

Organ transplant recipients are more prone to develop NMSCs because of their immunosuppression. In particular, cyclosporin A has been associated with higher risks of developing aggressive cSCCs and should be avoided in favor of sirolimus or everolimus, if feasible [129]. Unfortunately, data regarding the safety and efficacy of ICIs in organ transplant recipients are scarce and this population is usually excluded from this therapeutic option for the risk of graft rejection [23].

A recent review, based on 57 organ transplant recipients receiving ipilimumab, pembrolizumab, or nivolumab for solid tumors (melanoma in most cases) reported graft rejection in more than one-third of treated patients (37%, with death due to graft rejection in 14%) [130]. Kidney-transplanted patients were more susceptible to graft rejection compared to liver and heart transplant recipients. Nivolumab caused the most graft rejections (52%), whereas ipilimumab and pembrolizumab showed an inferior rate (25% and 26.7%) [130]. In another study by Abdel-Wahab et al. involving 37 patients (mostly suffering from melanoma), graft loss after ICIs therapy was 81% and death was reported in 46% of patients [131].

It is likely that time from transplantation and choice of immunosuppression could play a major role in defining the outcome in patients undergoing immunotherapy, although Abdel-Wahab et al. found no significative association between these factors [131].

There are case reports of organ transplant recipients with advanced NMSCs (cSCCs and MCCs) treated with ICIs experiencing both rejection [58,87,132] or preserved allograft function [91,95]. More research is needed in this respect and a phase I clinical trial is examining kidney transplant recipients with unresectable and advanced NMSCs who are treated with tacrolimus, prednisone, nivolumab, and ipilimumab (NCT03816332) [94].

#### 3.1.4. Patients Suffering from Autoimmune Conditions

According to a recent study involving 22 patients suffering from autoimmune conditions and treated for cancer (mostly melanoma and lung cancer) with ICIs, the results were encouraging [133]. Although de novo irAEs and the exacerbation of pre-existing autoimmune conditions were common, most patients tolerated ICIs therapy well [133].

### 3.2. Neutrophil to Lymphocyte Ratio and Other Serum Markers

Practical approaches to ICIs response prediction include the adoption of proxy indicators assessed on peripheral blood and other serum markers [134,135,136,137,138,139,140].

The neutrophil-to-lymphocyte ratio (NLR) is a marker for the general immune response to various stress stimuli and is considered an indicator of the tumor inflammatory micro-environment, which apparently correlates with the intratumor neutrophil population and the intratumor levels of myeloid-derived suppressor cells [134,135,136,137]. Moreover, a higher baseline NLR has been shown to associate with poor outcomes and responses to immunotherapy regardless of cancer type. Proposed cut-offs range from 3 to 5 [134,135,136]. Indeed, in patients treated with pembrolizumab for advanced MCC, a low neutrophil-to-lymphocyte ratio was associated with response and longer survival [84].

Other serum markers such as absolute lymphocyte count, leukocyte-to-lymphocyte ratio, monocyte count, eosinophil count, lactate dehydrogenase (LDH), circulating IgM-Rheumatoid, and circulating tumor DNA (ctDNA), as well as the presence of particular circulating cellular populations (like CD14+ CD16- HLA-DR^high^ monocytes [141] or PD-1+ Ki-67+ CD8+ T-cells [142]), have been investigated as predictors [137,138,139,140]. Moreover, elevated pretreatment serum levels of cytokines such as IFN-γ, interleukin (IL)-6, IL-8, and IL-10 have been proposed as potential positive predictors, while an increase in prolactin after immunotherapy treatment was associated to a poor response [143,144].

### 3.3. PD-L1 Expression: Histopathologic and Blood-Based Assessment

As a consequence of the growing role of immunotherapy, evaluating the expression of PD-L1 in the tumoral tissue seems a quite logical choice to assess its role as a therapeutic outcome predictor [139]. It is important to remember that PD-L1 is expressed on both neoplastic and tumor-infiltrating immune cells, including macrophages; on the other hand, PD-1 is expressed on CD8+ and CD4+ T-cells, B-cells, and natural killer (NK) cells [17,18,19,20,21]. PD-L1 expression is usually assessed on tumoral tissue with immunohistochemistry techniques and considered as a binary variable, which is scored as positive if present in more than 1% of tumor and tumor-infiltrating immune cells [17,18,19,20,21].

Notably PD-L1 is currently a United States FDA-approved biomarker for ICIs therapy, and it is generally believed that high PD-L1 expression is related to an increased response rate and clinical benefit in anti-PD-1/PD-L1 therapy for solid tumors [21,145,146]. However, the conclusions from multiple trials investigating this feature are not consistent and, moreover, this biomarker appears to be less promising in skin tumors [12,145,147]. For example, both high and low PD-L1 expression tumors demonstrated a gain in the clinical benefit from PD-1/PD-L1 blockades [12,41,148].

There is increasing interest in evaluating PD-L1 expression with simpler, minimally invasive techniques, which could be repeated multiple times during the course of the disease, thereby avoiding immunohistochemistry on tumoral tissue specimen [149]. A blood sample specimen suffices to evaluate many proposed proxy biomarkers, such as exosomes carrying PD-L1 mRNA or proteins (PD-L1+ exosomes), soluble PD-L1, or PD-L1+ circulating tumor cells [21,149]. The potential predictive role of these novel biomarkers has to be defined, but there is some evidence that the assessment of PD-L1+ exosomes could be useful in solid tumors (especially in melanoma) [150]. Interestingly, high levels of PD-L1+ exosomes before treatment initiation seem to represent a negative predictor because they indicate that the T-cells can no longer be reactivated by ICIs, denoting a sort of ‘immunological exhaustion’. On the contrary, an early increase in the level of PD-L1+ exosomes during treatment is a sign of T-cell reactivation and a better response to anti-PD-1 therapy [150]. In agreement with this, resistance to pembrolizumab was associated with a lack of increase in PD-L1+ exosomes in solid tumors [149]. This pattern was found in melanoma and NSCLC patients with complete and partial responses to nivolumab or pembrolizumab. In fact, PD-L1 mRNA levels in exosomes significantly declined from the start of treatment to the first re-evaluation, while a significant increase was observed in those who experienced progression [149].

Rescue of CD8+ T lymphocytes from PD-1/PD-L1 exhaustion has been shown to be CD28-dependent [151]. Indeed, the baseline expression of CD28 tends to be higher in the anti-PD-1 treatment responders with respect to the non-responders. A combined assessment of exosomal PD-L1 and CD28 could serve as a predictive tool for clinical responses to anti-PD-1 therapy and correlates with progression free survival [152].

Soluble PD-L1 (splicing variant of PD-L1 which lacks a transmembrane or intracellular domain) has a similar significance to PD-L1+ exosomes. Pre-treatment high levels are correlated with a worse response and a post-treatment increase is associated with a better outcome in melanoma and other solid tumors [153,154,155].

According to a study on 34 MCC patients, exosomal PD-L1 levels of MCC patients appear to be similar to those of healthy donors, but lower than those of melanoma patients. Although exosomal PD-L1 levels tended to be higher in MCC patients with distant metastases, they did not significantly vary over the course of the disease or the response to treatment [156].

On the other hand, PD-L1+ circulating tumor cells seem to have an opposite significance to PD-L1+ exosomes. As a matter of fact, patients with a high abundance of PD-L1+ cells at baseline tend to be sensitive to ICIs, while their reduction after treatment is related to a robust anti-tumor response [157].

In conclusion, the apparently contradicting results and the difficult interpretation of these data highlight that many mechanisms underlying tumoral immunology and tumoral response to immunotherapy have not been fully understood yet.

### 3.4. Tumor Infiltrating Lymphocytes and Tumoral Microenvironment

Tumor infiltrating lymphocytes (TILs) are a crucial component of the tumor micro-environment and it is proved that the activity of pre-existing TILs can be triggered by ICIs in solid tumors [158,159,160]. CD8+ TILs are supposed to play a major role in eliminating tumor cells directly and maintaining immune surveillance [21]. A model of the tumor micro-environment based on TILs status (presence/absence) combined with the PD-L1 expression status has been proposed to immunologically categorize different cancers and predict their response to immunotherapy: PD-L1+ TIL+ tumors are most likely to respond to PD-1/PD-L1 blockade therapy [161]. Evaluating TILs through histological examination is considered the gold standard, but recently the assessment of TIL transcriptome (RNA expression) has gained great interest [162]. Through RNA sequencing analysis, it is possible to look for specific TIL signatures that can be considered pan-cancer predictive biomarkers of anti-PD-1 response [162].

With regard to the tumoral micro-environment, high levels of transforming growth factor β (TGF-β) produced by peritumoral fibroblasts seem to hinder immune response, hampering T-cell migration in tumor stroma [163]. A TGF-β signature has been related with non-response to ICIs and tumor progression [163]. Conversely, high levels of interferon (IFN)-γ seem to correlate with clinical benefits from anti-PD-1 treatment in patients with head and neck SCC and other solid cancers [145,164]. IFN-γ has antiproliferative and immunostimulatory activity but can act as a double-edged sword. In fact, constant exposure to this cytokine during anti-PD-1/PD-L1 treatment can lead to adaptive resistance to immunotherapy [164,165].

### 3.5. Genetic Alterations: Tumor Mutational Burden and Copy-Number Alteration

It is known that all cancers with a high tumor mutational burden (TMB), which is the number of DNA mutations in malignant cells, respond well to immunotherapy [8,146]. The higher the TMB, the more significant the tumor antigenicity, meaning that the malignancy can become an easy target for the patient’s immune system [8,9,10]. However, a TMB analysis is quite expensive, and this fact could restrict its clinical applications [166].

The advent of Next Generation Sequencing (NGS) and whole exome sequencing techniques allows for the investigation of the mutational profile of different neoplasms with the aim of having a better understanding of the pathogenetic mechanisms of these diseases [17]. With regard to the genetic alterations of tumoral cells, copy-number alterations (CNAs, specific changes in chromosome structure resulting in deletion or amplification of genome portions) have recently been shown to correlate with gene signatures of immune evasion and worse survival in response to CTLA-4 blockade [167]. According to recent research, high TMB and low CNAs represent two independent predictors of response to ICIs treatment in different solid tumors; therefore, these parameters can jointly stratify cancer patients into groups with different prognosis and clinical responses to immunotherapy [166]. Unfortunately, although involving a large number of solid tumors, this research did not include NMSCs in the pool of analyzed malignancies [166]. TMB and CNAs could surely represent useful predictors, but other mechanisms are likely to play a role in defining the response to immunotherapy and further research is needed to understand the complexity of this phenomenon.

In the near future, a better understanding of gene expression signatures in tumoral tissue together with the increasing availability of RNA sequencing techniques will allow for finding new predictors in solid tumors. In this context, dealing with huge amounts of data, machine learning analysis, and artificial intelligence will undoubtedly represent a promising approach to identify relevant predictive biomarkers [168,169].

## 4. cSCC and BCC Immunobiology and Tumor-Specific Predictors

Cutaneous squamous cell carcinomas (cSCCs) and basal cell carcinomas (BCCs) originate from epidermal keratinocytes. Chronic exposure to UV radiation is a well-known risk factor for these cancers and also a strong mutagen, therefore it is not surprising that they show the highest tumor mutational burden (TMB) among all cancer types (about 47.3 and 45.2 mutations/Mb, respectively)—along with virus negative-Merkel cell carcinomas (VN-MCCs) [170]. This high TMB increases immunogenicity, as tumoral cells express neoantigens that can be recognized by immune cells [170]. As a matter of fact, BCCs, and to a lesser extent cSCCs, are known to express cancer-testis antigens (CTAs), which are normally only detected in trophoblastic and male germline cells. This phenomenon could be due to the demethylation of the promoter of specific genes [171].

However, the high TMB does not explain the complexity of the immunological asset of these NMSCs. Despite its higher TMB, there is some evidence that BCC has a reduced antigen presentation compared with cSCC, possibly leading to an overall lower tumor immunogenicity [11]. This phenomenon could be due to the downregulation of proteins involved in antigen modification and presentation, such as transporters associated with antigen processing-1 (TAP-1) and major histocompatibility complex I (MHC-I), but could also be due to diminished infiltration by CD4+ and CD8+ T-cells with an increased presence of regulatory T-cells (T-regs), and immunosuppressive effects driven by IL-10 and Th2 cytokines [11].

In addition, BCC and cSCC differ in terms of infiltrating immune cells. The peri- and intratumoral infiltration of CD8+ T-cells is drastically reduced in BCC compared with cSCC, and this aspect could be linked to the abovementioned downregulation of MHC-I [170]. These observations led to the hypothesis that cSCC could be more prone to immune surveillance than BCC. Indeed, in immunosuppressed patients, the risk of developing squamous cell carcinomas increases more compared to basal cell carcinomas [172].

T-regs can be found both in BCCs and cSCCs. These cells are known to inhibit the proliferation of both CD4+ and CD8+ T-lymphocytes and IFN-γ release; moreover, a significative presence of immature dendritic cells has been reported in the stroma of BCC, contributing to an immunosuppressive micro-environment [173,174,175]. Cytokines such as TGF-β, IL-4, IL-6, IL-1B, and IL-10 have been reported to be upregulated in BCCs; notably, the immunosuppressive cytokines, TGF-β and IL-10, are overexpressed in BCCs and in high-grade cSCCs, whereas low-grade cSCCs show a lower expression [176,177].

In summary, BCC and cSCC display different immunological assets, and BCC appears to be less subject to immune surveillance. Immunotherapy has been shown to be useful in cSCC and its potential efficacy in reversing BCC immune escape is a recent acquisition [12]. Validated predictors of response to ICIs specific to cSCC are not yet available and the literature regarding BCC is extremely scarce in this regard. Possible predictive parameters evaluated so far for cSCC and BCC will be reviewed below and are summarized in Table 7.

### 4.1. Clinical Predictors

According to a study by In et al. involving 26 patients with advanced cSCCs treated with cemiplimab, pembrolizumab, or nivolumab, a primary tumor location on the head/neck was associated with a response to PD-1 inhibition (*p*-value = 0.04) [42]. In this research, other clinical features (including age, gender, immune suppression, performance status, previous treatments, tumor high-risk features, or disease burden) were not related to response [42]. Conversely, Saltzmann et al. found that patients with primaries located on the leg had poorer therapeutic outcomes with PD-1 inhibitors [178]. Notably, in a recent retrospective study involving 61 patients with advanced cSCC, Hanna et al. investigated numerous clinical parameters as predictors of response to cemiplimab, pembrolizumab, and nivolumab. A primary tumor location on the head/neck was not confirmed as a positive predictor and again no association with response was found for age, gender, performance status, smoking history, immune suppression, initial staging, pathologic differentiation of tumor, initial treatment regimen, disease burden, and ICIs line of therapy [179]. In contrast, an association between head/neck location and longer PFS (but not OS) has been documented in a larger, real-life French study, including 245 patients with la-cSCC or m-cSCC treated with cemiplimab. The same study revealed an association between performance status and both PFS and OS [44]. A smaller real-world study on 18 cemiplimab-treated patients with la-cSCC or m-cSCC demonstrated an association between response and age above 60 years [43]. Another real-world retrospective study involving 131 patients with advanced cSCC treated with cemiplimab identified a head and neck tumor location (*p*-value = 0.007) and normal haemoglobin values (*p*-value = 0.034) as positive predictors; on the other hand, location on the genitalia (*p*-value = 0.041), treatment with any systemic antibiotic within 1 month of cemiplimab initiation (*p*-value = 0.012), performance status ≥1 (*p*-value = 0.012), chronic corticosteroids therapy (*p*-value = 0.038), previous radiation therapy to lymph nodes (*p*-value = 0.052), and previous chemotherapy (*p*-value = 0.0020) were significantly associated with a worse response [45]. In a cohort of 30 elderly frail patients with la-cSCC (*n* = 25) or m-cSCC (*n* = 5) treated with cemiplimab, head/neck tumor location (*p*-value = 0.016) and haemoglobin > 12 g/dL (*p*-value = 0.042) were similarly associated with a higher RR [51].

### 4.2. Absolute Lymphocyte Count (ALC) and Other Serum Markers

Absolute lymphocyte count (ALC) was investigated as a predictor of response to ICIs in the aforementioned retrospective study by Hanna et al., and a higher median ALC at therapy initiation was correlated with a good response (*p*-value < 0.01) [179]. Strippoli et al. also identified the association between baseline low neuthophil/lymphocyte ratio and low platelet/lymphocyte ratio as a serological predictor of better response [51]. The underlying biological hypothesis is that lymphocyte count may serve as a surrogate for estimating the number of available circulating lymphocytes that could target the tumor, thus contributing to immunosurveillance [179].

As far as other serum markers are concerned, IL-6 and IL-8 cytokine levels have been associated with tumor development and progression, and their role has been well-documented in a wide range of tumors, including NMSCs. Therefore, they could potentially be failure-predicting biomarkers for ICIs treatment [145]. Saltzmann et al. demonstrated poorer therapeutic outcomes in cSCC patients with elevated lactate dehydrogenase serum levels at baseline [178].

### 4.3. PD-L1 Expression

Several studies investigated the role of PD-L1 expression in cSCC. Generally, PD-L1 expression was assessed on immunohistochemistry, with ≥1% staining defining positive cases [41,52,65,180,181,182,183]. In cSCC, higher PD-L1 expression has been associated with aggressive, high-risk tumors [181,183] and also poor prognosis [182], but these works preceded the advent of immunotherapy.

According to a recent report of the CARSKIN phase II trial involving patients with unresectable cSCC treated with pembrolizumab, the overall RR at 15 weeks for the 42 patients with PD-L1+ tumors at baseline (55%) was significantly higher than for the 12 patients (17%) with PD-L1- status (*p*-value = 0.02). Only two responders presented PD-L1- cSCCs [65]. These results must be interpreted cautiously because PD-L1 analysis was exploratory and unadjusted [65].

A similar trend was observed in a recent analysis of 48 patients with la-cSCC treated with cemiplimab. In fact, although the therapeutic regimen was highly active in both PD-L1 positive and negative subgroups, the overall RR was higher for PD-L1+ patients (54.8% vs. 35.3%) [41]. Of note, PD-L1 levels appear to be similar in metastases when compared with their primary tumors, but there are cases with a non-concordant expression and a tendency toward an increase in distant localizations; this eventuality could have implications for treatment management [184].

As far as BCC is concerned, PD-L1 expression did not correlate with response to cemiplimab according to recent data on 50 patients with la-BCC [12]. Objective RR was 26% (95% CI: 13–43%) in 35 individuals with PD-L1 expression < 1% and 27% (95% CI: 8–55%) in 15 patients with PD-L1 expression ≥ 1% [12]. These findings confirmed previous reports by Chang et al. [75].

### 4.4. Genetic Alterations: Tumor Mutational Burden and Copy Number Alterations

TMB was investigated as a predictor of response to ICIs (cemiplimab, pembrolizumab, and nivolumab) in the abovementioned study by Hanna et al. involving 61 patients with advanced cSCC [179]. In this research, higher median TMB values were observed among responders (25.4 versus 10.6 mutations per Megabase [muts/Mb], *p*-value = 0.02) [179]. A recent analysis of a cohort of 50 patients with advanced cSCC treated with cemiplimab confirmed this tendency. Among 29 patients who achieved durable disease control and 21 patients who did not, the median TMB was 64.9 and 31.5 muts/Mb, respectively [41]. Moreover, in this cohort, an association between high TMB and 12-month PFS and OS was also recognized [41]. Similar results were reported by In et al., who considered 26 patients with advanced cSCCs treated with anti-PD-1 (cemiplimab, pembrolizumab, or nivolumab). The median TMB was higher among responders compared to non-responders (60 vs. 9 muts/Mb, *p*-value = 0.04) [42].

However, as previously anticipated, the genomic landscape of cSCC appears to be more complex. Preliminary findings from tumor genomic analysis also revealed that low-level copy number alterations (CNAs) in the 3q chromosomal arm (bands 21–27, a region that includes *ETV5*, *PIK3CA* and *BCL6* genes) are associated with favorable therapeutic outcomes (*p*-value < 0.01) [119].

## 5. MCC Immunobiology and Tumor-Specific Predictors

Merkel cell carcinomas (MCCs) can be subdivided into virus-positive (VP-MCCs) and virus-negative tumors (VN-MCCs), depending on the clonal integration of Merkel cell polyomavirus (MCPyV) in the DNA of tumoral cells [185]—whose role was first described in 2008 [185,186]. MCPyV is a small, double-stranded DNA virus that is normally present in cutaneous flora, but in a fraction of people it integrates into the cellular genome, leading to MCPyV T-antigen oncoprotein expression and causing an uncontrolled growth of the transformed cells [187]. Almost 80% of MCC cases in the United States and Europe are classified as VP-MCCs, while the remainder lack detectable tumor-associated MCPyV DNA or oncoproteins and can be classified as VN-MCCs [11,185]. This proportion of VN-MCCs is more represented in Australia and these cancers show a UV mutational signature [188,189].

Despite having a low mutational burden, VP-MCCs express viral proteins that can be recognized by the humoral and cellular arms of the immune system [185,190]. On the other hand, VN-MCCs show a high mutational burden due to chronic UV exposure, which likely leads to the generation and expression of novel proteins and epitopes acting as immunogenic neo-antigens [188]. However, MCCs are known to evade immune responses, downregulating antigen presentation and the expression of major histocompatibility complex I (MHC I) [191]. They can also induce an immunosuppressive tumoral micro-environment, producing immunosuppressive cytokines and recruiting immunosuppressive cells, such as CD4+ CD25+ regulatory T-cells (T-regs) or myeloid-derived suppressor cells [192]. Due to chronic antigen exposure, antigen-specific CD8+ T-cells in a tumoral micro-environment commonly develop an exhausted phenotype with reduced activity, which is characterized by the expression of inhibitory receptors such as PD-1 and TIM-3 [190].

All these observations provided a strong rationale for investigating PD-1/PDL-1 blockade in MCCs, leading to a dramatic change in the therapeutic landscape of these tumors. Indeed, immunotherapy can lead to good results in both VN-MCCs and VP-MCCs subtypes [11]. According to a recent systematic review including 6 clinical trials and 201 individual patients, similar response rates are achieved regardless of viral status or programmed death ligand-1 expression, suggesting that immunotherapy might act on multiple, unexplored pathways [193].

Although very promising, even ICIs are not foolproof. A non-response rate of approximately 50% to first-line treatment with PD-1/PD-L1 inhibitors has been documented [186]. Possible predictive parameters evaluated so far for MCC will be reviewed below and are summarized in Table 8.

### 5.1. Clinical Predictors and Laboratory Biomarkers

In the selection process of candidates for immunotherapy among patients with MCC, some features have been proposed as clinical predictors, these include performance status, immunosuppression, and number of previous lines of treatment [17,19]. In patients treated with ICIs, a better overall performance status and absence of immunosuppression were associated with response to therapy [19]. Moreover, in a recent study of Knepper et al., response rate was significantly correlated with line of therapy, with 75% of ICIs being administered as first-line, 39% as second-line, and 18% as third-line or beyond [17].

According to the results of the KEYNOTE-017 trial in 50 patients on pembrolizumab for unresectable MCC, a baseline Eastern Cooperative Oncology Group (ECOG) performance status of 0 and completion of 2 years of treatment were associated with response and longer survival [84]. An even larger retrospective study including 114 MCC cases revealed that the absence of immunosuppression and a limited number of tumor-involved organ systems were highly associated with a favorable response. The same research highlighted that unimpaired performance status, advanced age, normal LDH, and normal C reactive protein are moderately associated with disease control [194].

In a very recent real-world study involving 20 patients with advanced MCC treated with avelumab, a body mass index (BMI) ≥ 30 was significantly associated with longer time to treatment failure (*p*-value = 0.004) and objective RR (*p*-value = 0.01). This finding extends the concept of the “obesity paradox” and the role of BMI as a predictive factor for ICIs therapy [106].

MCC is a neuroendocrine neoplasm and, accordingly, neuron-specific enolase (NSE) has been suggested as a possible biomarker. Although baseline NSE had no association with prognosis in a study of 84 patients, during immunotherapy (*n* = 23) all those experiencing a CR (*n* = 10) had normalized NSE (< 18.2 ng/mL) values, all those achieving a PR (*n* = 5) had decreasing NSE levels, while all non-responders (*n* = 8) had persistently elevated NSE concentrations [196].

### 5.2. MCPyV-Specific B-Cells and T-Cells Activity

MCPyV-specific T-cells and circulating antibodies against MCPyV T-antigens have been correlated with disease burden and have been shown to decrease after treatment with surgery or radiation [200]. Miller et al. analyzed MCPyV-specific B-cell and T-cell activity at baseline and throughout treatment in VP-MCCs treated with pembrolizumab [195]. B-cell activity was defined by the titer of circulating antibodies against MCPyV oncoprotein T-antigen. T-cell activity was evaluated by isolating tumor-specific CD8+ T-cells and measuring the production of cytokines (IFN-γ and IL-2) by CD8+ and CD4+ T-cells challenged with MCPyV peptides [195]. According to this study, specific B-cell and T-cell activity were correlated with disease burden and may be useful to identify early recurrences in MCPyV-positive MCCs, but could not be used as a predictor of response to ICIs before treatment [195].

### 5.3. PD-L1 and PD-1 Expression and Density, PD-L1/PD-1 Proximity and TILs

Several authors analyzed tumoral PD-L1 expression in MCC as a potential predictive biomarker of response to immunotherapy. This parameter was usually evaluated on immunohistochemistry or pre-treatment biopsy specimens, and was generally classified as positive in the presence of more than 1% of tumor cells staining for PD-L1 [17,18,19,20]. These investigations were prompted by the observation that PD-L1 expression on tumor cells and on CD8+ TILs had been previously found to be associated with an improved survival in advanced MCC [201,202,203]. In some cases, PD-L1 expression was assessed on cells at the periphery of tumor nodules, since PD-L1 has been shown to be more present there [17]. Nighiem et al. evaluated PD-L1 expression on tumoral cells and TILs [18]. Similarly, Spassova et al. determined PD-L1 on tumoral tissue, however, tumor-infiltrating inflammatory cells and other stromal cells were excluded from this evaluation [19].

Despite slight differences in the assessment of the parameters, all the studies agreed on the conclusion that PD-L1 expression does not configure as a reliable biomarker of response to ICIs [17,18,19,20,83]. It is noteworthy that a slight tendency of improved PFS and OS was shown in patients carrying PD-L1 in a pre-treatment setting and receiving pembrolizumab [18]. A similar result was found in patients with chemotherapy-refractory MCCs receiving avelumab (cohort A of JAVELIN Merkel 200 study) [20,103]. In addition, in a real-world study, preliminary data analysis on 6 tumoral and plasma samples suggested that serum soluble PD-1 > 3.8 ng/mL, and the presence of PD-L1 and brisk TILs on tumor samples, were associated with a longer time to treatment failure [106].

Moreover, one of the abovementioned studies also determined PD-1 expression on peritumoral lymphocytes through immunohistochemistry (within a distance of 20 μm from tumor) in 27 MCC treated with ICIs [17]. Due to the relative paucity of PD-1 expression, the staining was scored solely on the basis of presence/absence of its expression. Interestingly, there was a statistically significant association between PD-1+ status and RR, with response to ICIs in 10 of 13 (77%) PD-1 positive patients, compared with 3 of 14 (21%) PD-1 negative subjects (*p*-value = 0.00598) [17].

Giraldo et al. performed a more complex analysis of the tumoral micro-environment in MCCs, with the aim to understand its influence on response to pembrolizumab [197]. The study consisted of an expanded histopathologic analysis on pre-treatment tumor samples using immunohistochemistry/immunofluorescence aided by next-generation digital imaging techniques [197]. In particular, topographic quantitative and spatial proximity analyses were performed on CD8+, PD-1+, and PD-L1+ cell populations to assess their density, distribution, and proximity [197]. The study concluded that there was no significant association between CD8+ TILs density and clinical response; on the other hand, tumors from patients who responded to ICIs showed higher densities of PD-1+ and PD-L1+ cells when compared to non-responders (median cells/mm^2^, 70.7 vs. 6.7, *p*-value = 0.03; and 855.4 vs. 245.0, *p*-value = 0.02, respectively). Notably, cell types expressing PD-1 included CD8+ T-cells, CD4+ T-cells, T-regs, and CD20+ B-cells, supporting the notion that multiple cellular subtypes may be involved in tumor response to ICIs. In addition, in this study the authors also performed the assessment of both CD8/PD-L1 and PD-1/PD-L1 proximity, representing the contiguity of cells that express these markers—considering a maximum distance of 20 μm. Interestingly, PD-1/PD-L1 proximity was associated with clinical response (*p*-value = 0.03), while CD8/PD-L1 proximity was not [197]. The concept of proximity appears very promising because taking into account the spatial distance between PD-1 and PD-L1 eliminates the bias given by the mere quantification of the individual parameters. Although these molecules are expressed in large quantities, they may not interact as expected due to being overly spatially distant. This would explain at least some unsatisfactory responses. Thus, a distance assessment between these markers (i.e., proximity) may offer a better estimation of their interaction [197].

In the recently published results of the JAVELIN Merkel 200 study, response rates tended to be numerically higher in patients with PD-L1+ tumors and those with increased intratumoral CD8+ T-cell density [100]. Concordantly, Spassova et al. demonstrated the predominance of CD8+ effector and central memory T-cells in close proximity to tumor cells in patients with a favorable response to PD-1/PD-L1 blockade [194].

Recently, a study analyzing TILs in 58 MCC lesions (belonging to 39 patients) characterized a previously unidentified population of γδ T-cells. Interestingly, γδ T cell-enrichment correlated with longer disease-specific survival, with 3 out of 4 of such γδ T cell-enriched MCC patients demonstrating CR to anti-PD-L1 treatment [198].

Finally, a study harnessing the power of a new method for single-cell cytometry investigations, FAUST, uncovered a population of effector memory CD4+ and CD8+ T-cells co-expressing CD28, HLA-DR, and PD-1 in the peripheral blood of MCC patients, which may be a candidate biomarker for response to pembrolizumab. These findings are also in line with the well-known importance of CD28 expression in CD8+ T-cells during anti-PD1 immunotherapy [199].

### 5.4. TILs TCR Repertoire in Pre-Treatment Tumors

T-cell receptor (TCR) clonality can be assessed using techniques of genomic DNA extraction from formalin-fixed, paraffin-embedded (FFPE) tumor biopsy before starting treatment [195]. Increased clonality is associated with reduced T-cell diversity and identifies a cellular response restricted to a few antigens; on the other hand, low clonality corresponds to a T-cell population that is active against multiple antigens or distinct clones specific to the same antigen [195].

The idea to assess TCR clonality as a predictor of response to immunotherapy came from the evidence that, in metastatic melanoma, a higher clonality was associated to a better response to pembrolizumab [204]. This finding was considered representative of specific T-cell response against neoplastic antigens [204]. Regarding MCC, it has been observed that TCR clonality is greater in MCPyV-positive neoplasms, corresponding to the expansion of a limited number of clones in response to specific MCPyV antigens [195]. Conversely, MCPyV-negative MCCs are characterized by lower clonality, identifying a T-cell response to numerous neoantigens [198].

Spassova et al. performed an analysis on 41 real-life setting MCC patients, focusing on clinical, molecular, and immunological features [19]. A parameters assessment was conducted before starting ICIs and a subsequent determination was made after starting therapy, if possible, with the aim of identifying predictive biomarkers of response to treatment [19]. The molecular work-up included TCR clonotype sequencing, multiplexed immunofluorescence staining, and immune gene mRNA expression analysis of TILs performed on FFPE tissue samples. It emerged that functional characteristics of TILs correlated with response to ICIs. In particular, responders showed a predominance of central memory T-cells, with low T-cell clonality and high TCR diversity. Conversely, non-responders were characterized by low TCR diversity, identifying terminally differentiated effector T-cells with impaired proliferative capacity [19]. Moreover, in the same study, a panel of approximately 100 genes involved in adaptive immunity, lymphocyte activation, leukocyte migration, and cytokine signaling pathways were analyzed. MCC of responders expressed genes related to T-cell attraction (CCL5, CXCL9, IL16, CXCL11, CCL3, CXCL10, CCL21, and CCL4) or activation (IL2RB, IL2RG, IL15RA, LCK, CD97, JAK3, and NFATC2). Conversely, the expression of these genes was low or absent in non-responders, which instead showed a strong expression of CDK1 and BCL2 genes [19].

### 5.5. Genetic Alterations: Tumoral Mutational Burden and MCPyV Status

Performing a comprehensive genomic profile on MCCs is one of the strategies adopted to find possible biomarkers correlating with response to therapy [17]. It is well known that VP-MCCs are associated to a low TMB, while VN-MCCs appear to be ultraviolet (UV)-driven malignancies, which are characterized by a high TMB [17,205,206]. The UV-signature is mainly characterized by C (cytosine) > T (thymine) transition mutations in a CC or TT dinucleotide context [17,206]. In MCC, these mutations often concern KMT2C/D, FAT1, and LRP1B genes, which is similar to cSCC; instead, mutations in TP53 and RB1 appear to be very frequent in both VN and VP subgroups [17,205,206].

Different research teams focused on MCPyV status as a possible predictor of response, determining it through various methods. In particular, Knepper et al. identified MCPyV presence by using the NGS method to extract viral DNA embedded in neoplastic cells, which obtained FFPE specimens of MCCs biopsies; an additional investigation consisting of the detection of viral antigens on tumor tissue samples by IHC (immunohistochemistry) was performed when available [17]. Another technique for detecting viral status consisted of performing PCR analysis on tissue biopsy samples [19]. Nghiem et al. identified MCPyV status through the determination of small T-antigen-specific antibodies in patients’ serum and the identification of large T-antigen expression in tumor biopsies via immunohistochemistry (IHC) [18]. Putting together all these findings, it results that current evidence does not qualify MCPyV status as a solid predictor of response. Indeed, MCC appears to be highly responsive to anti-PD-1 therapy, regardless of viral status [17,18,19]. It can be assumed that the response pattern to ICIs follows different but equally effective mechanisms in both subtypes of MCC. Anyway, it must be reported that in the JAVELIN Merkel 200 study, response rates were numerically higher in patients with MCPyV-negative MCCs [100].

As mentioned above, studies carried out on other types of neoplasm showed that, in general, ICIs provide greater clinical benefit to those with higher TMB, probably because of the increased antigenicity conferred by the mutations [186]. This mechanism could be true for VN-MCC as well, being characterized by a higher TMB. Conversely, in VP-MCC an immunological escape process may be prominent. In fact, an upregulation of PD-1 in tumor infiltrating and peripheral blood MCPyV-specific T-cells has been demonstrated in VP-MCC, leading to the suppression of immune responses against the neoplasm [17,186]. Immunotherapy with PD-1/PD-L1 inhibitors appears to be effective for these cancers because it reverses this mechanism [186]. Moreover, the satisfactory response to ICIs of VP-MCC may also imply that viral antigens themselves also constitute a source of immunogenicity [186].

## 6. Conclusions

In summary, most guidelines consider ICIs as a potential treatment strategy for locally advanced or metastatic NMSCs in a first-line setting or in HHI-resistant BCCs. Despite intense research, evidence is still limited for predictors of ICIs response.

From a practical point of view, the selection of candidates deserving ICIs should be balanced on an accurate validation of benefits and risks in a multidisciplinary setting. In particular, there is emerging evidence that elderly individuals [120,121] and patients suffering from autoimmune conditions [133] could benefit from immunotherapy, whereas in organ transplant recipients, ICIs use should be carefully evaluated because of the risk of possible organ rejection [130,131].

With regard to cSCC, a higher TMB [41,179], genetic alterations in the 3q chromosomal arm [119], and PD-L1+ status [41,65] show a tendency to be associated to positive clinical responses. A more simple and reproducible approach is represented by serum biomarkers, such as absolute lymphocyte count [179], but their role should be confirmed by further studies. Supportive data for BCC are even more scarce, probably because immunotherapy use is less common for this tumor.

Regarding MCC, while MCPyV status [17,18,19,186] and PD-L1+ expression (≥1%, evaluated with immunohistochemistry) [17,18,19,20,83] cannot be considered useful predictors of response, PD-1 and PD-L1 density, PD-L1/PD-1 proximity [197], and features of TILs TCR repertoire (with low clonality and high diversity) [19,195] are associated with good outcomes.

It is necessary to underline that many of these proposed biomarkers are assessed by employing complex, expensive, and not easily reproducible genetic, immunohistochemistry, or immunofluorescence techniques that are potentially difficult to use in daily practice and not cost-effective. However, if available, they can offer a more comprehensive picture, complementing the patient’s clinical data. Identifications of potential responders and non-responders could be useful to customize treatment choice in different patients with a tailored approach.

In conclusion, a better future comprehension of the mechanisms of response to therapy may be helpful in framing possible predictors for ICIs therapy. Definitive, cost-effective, and reproducible biomarkers are still lacking, and further efforts are needed to validate the suggested predictors in larger cohorts.

## Figures and Tables

**Table 1:** Reported cases of locally advanced and metastatic non-melanoma skin cancers treated with ipilimumab.

Number of Patients	Patient Data (Sex, Age)	Diagnosis	Ipilimumab Regimen	Outcome	Additional Information	Ref.
1	M, 72 y	m-cSCC	3 mg/kg ipilimumab IV every 3 weeks	PR	Concurrent melanoma	Day et al., 2017 [28]
1	M, in his 60 s	la-BCC	3 mg/kg ipilimumab IV every 3 weeks	PR	Concurrent melanoma	Mohan et al., 2016 [29]
5	4 M, 1 F; age: 50–81 y	m-MCCs	3 mg/kg ipilimumab IV every 3 weeks	2 CR; 2 SD; 1 PD	/	Winkler et al., 2017 [31]
5	4 M, 1 F; age: 57–70 y	m-MCCs	3 mg/kg ipilimumab IV + nivolumab 1 mg/kg IV OR 1 mg/kg ipilimumab IV + nivolumab 3 mg/kg IV	1 CR; 2 PR	Avelumab refractory disease	Glutsch et al., 2021 [36]
1	M, 65 y	m-MCC	1 mg/kg ipilimumab IV every 6 weeks + 240 mg nivolumab IV. every 2 weeks	CR	Avelumab refractory disease	Khaddour et al., 2021 [37]

Note: Abbreviations: M (male); F (female); y (years); m-cSCC (metastatic cutaneous squamous cell carcinoma); la-BCC (locally advanced basal cell carcinoma); m-MCC (metastatic Merkel cell carcinoma); IV (intravenously); CR (complete response); PR (partial response); SD (stable disease); PD (progressive disease).

**Table 2:** Reported cases of locally advanced and metastatic non-melanoma skin cancers treated with cemiplimab.

Number of Patients	Patient Data (Sex, Age)	Diagnosis	Cemiplimab Regimen	Outcome	Additional Information	Ref.
Cohort I: 26 Cohort II: 59	Cohort I: 21 M, 5 F; median age: 73 y Cohort II: 54 M, 5 F; median age: 71 y	Cohort I: 10 la-cSCCs; 16 m-cSCCs Cohort II: 59 m-cSCCs	3 mg/kg cemiplimab IV every 2 weeks	Cohort I: 0 CR (0%); 13 PR (50%); 6 SD (23%); 3 PD (12%) Cohort II: 4 CR (7%); 24 PR (41%); 9 SD (15%); 11 PD (19%)	EMPOWER-CSCC-1 trial (phase I/II study). This trial led to drug approval for la-cSCC and m-cSCC	Migden et al., 2018 [38]
78	59 M, 19 F; median age: 74 y	la-cSCCs	3 mg/kg cemiplimab IV every 2 weeks	10 CR (12.8%); 24 PR (30.8%); 28 SD (35.9%); 9 PD (11.5%)	Phase II cohort from EMPOWER-CSCC-1 trial	Migden et al., 2019 [41]
13 (in a cohort of 26 patients)	Data for the entire cohort: 19 M, 7 F; median age: 64.5 y	Data for the entire cohort: 5 la-cSCCs; 21 m-cSCCs	Not specified	RR 46.2%	The entire cohort included 26 patients treated with cemiplimab (13 patients), pembrolizumab (7) and nivolumab (6)	In et al., 2020 [42]
18	16 M, 2 F; median age 80 y	9 la-cSCCs; 5 m-cSCCs	3 mg/kg cemiplimab IV every 2 weeks or 350 mg IV every 3 weeks	Overall RR 67%; 6 CR (33%); 6 PR (33%); 1 SD (6%); 5 PD (28%)	/	Guillaume et al., 2021 [43]
245	178 M, 73 F; mean age 77 y	35% la-cSCCs; 65% m-cSCCs	3 mg/kg cemiplimab IV every 2 weeks	Best overall RR 50%; CR 21%; PR 29%, SD 9%; PD 41%	Real-life experience from the French CAREPI Study Group	Hober et al., 2021 [44]
131	90 M, 41 F; median age 79 y	91 la-cSCCs; 40 m-cSCCs	350 mg IV every 3 weeks	Overall RR 58%; CR 21 (16%); PR 55 (42%), SD 18 (13.7%); PD 31 (23.7%)	Real life data from Italian multicenter study	Baggi et al., 2021 [45]
30	24 M, 6 F; median age 81 y	25 la-cSCCs; 5 m-cSCCs	350 mg IV every 3 weeks	Overall RR 76.7%; CR 9 (30%); PR 14 (46.7%), SD 1 (3%); PD 6 (20%)	Cohort of elderly frail patients	Strippoli et al., 2021 [51]
2	F, 66 y; M, 52 y	m-BCC; m-cSCC	10 mg/kg cemiplimab IV every 2 weeks; 1 mg/kg cemiplimab IV every 2 weeks	1 PR; 1 CR	/	Falchook et al., 2016 [47]
2	Not specified	la-BCCs	1–10 mg/kg cemiplimab IV every 2 weeks ± hfRT ± cyclophosphamide	1 PR; 1 SD	Research conducted on 60 patients with different advanced solid cancers	Papadopoulos et al., 2020 [50]
84	56 M, 28 F; median age: 70 y	la-BCCs, patients resistant or intolerant to HHI	350 mg cemiplimab every 3 weeks	5 CR (6%); 21 PR (25%); 41 SD (48.8%); 9 PD (10.7%)	NCT03132636 trial (phase II study) that has granted FDA approval for cemiplimab in la-BCC and m-BCC, resistant or intolerant to HHI	Stratigos et al., 2021 [12]
1	M; 80 y	la-cSCC + la-BCC	350 mg cemiplimab every 3 weeks	CR for both lesions	/	Dumann et al., 2021 [48]
1	M, 78 y	la-BCC	350 mg cemiplimab every 3 weeks	CR	Patient included in NCT03132636 trial	De Giorgi et al., 2021 [49]
2	Not specified	la-MCCs	3 mg/kg cemiplimab every 2 weeks + hfRT	1 PR; 1 SD	Research conducted 60 patients with different advanced solid cancers	Papadopoulos et al., 2020 [50]

Note: Abbreviations: M (male); F (female); y (years); la-cSCC (locally advanced cutaneous squamous cell carcinoma); m-cSCC (metastatic cutaneous squamous cell carcinoma); la-BCC (locally advanced basal cell carcinoma); m-BCC (metastatic basal cell carcinoma); la-MCC (locally advanced Merkel cell carcinoma); IV (intravenously); HHI (hedgehog inhibitor); hfRT (hypofractionated radiotherapy); FDA (Food and Drug Administration); CR (complete response); PR (partial response); SD (stable disease); PD (progressive disease); RR (response rate).

**Table 3:** Reported cases of locally advanced and metastatic non-melanoma skin cancers treated with pembrolizumab.

Number of Patients	Patient Data (Sex, Age)	Diagnosis	Pembrolizumab Regimen	Outcome	Additional Information	Ref.
2	M, 79 y; M 65 y	m-cSCCs	2 mg/kg IV every 3 weeks	1 PR; 1 SD	One of the patients (65 y, SD) was HIV positive	Borradori et al., 2016 [56]
1	M, 67 y	m-cSCC	2 mg/kg IV every 3 weeks	CR	/	Assam et al., 2016 [54]
1	M, in his 70 s	la-cSCC	2 mg/kg IV every 3 weeks	PR	/	Chang et al., 2016 [57]
1	F, 57 y	m-cSCC	Not specified	PR	Patient was kidney transplant recipient and ICI therapy caused allograft rejection	Lipson et al., 2016 [58]
1	M, 74 y	la-cSCC	Not specified	PR	/	Winkler et al., 2017 [59]
5	3 M, 2 F; median age: 72.6 y	m-cSCCs	2 mg/kg IV every 3 weeks	1 CR; 3 PR; 1 PD	/	Tran et al., 2017 [53]
1	M, 48 y	m-cSCC	2 mg/kg IV every 3 weeks	PR	Patient with xeroderma pigmentosum	Deinlein et al., 2017 [60]
1	M, in his late 70 s	la-cSCC	2 mg/kg IV every 3 weeks	PR	/	Stevenson et al., 2017 [52]
1	M, 63 y	m-cSCC	2 mg/kg IV every 3 weeks	CR	Patient was kidney transplant recipient and ICI therapy did not cause allograft rejection	Sadaat et al., 2018 [55]
2	M, 80 y; M, 76 y	la-cSCCs	2 mg/kg IV every 3 weeks	2 PR	/	Degache et al., 2018 [61]
7 (in a cohort of 26 patients)	Data for the entire cohort: 19 M, 7 F; median age: 64.5 y	Data for the entire cohort: 5 la-cSCCs; 21 m-cSCCs	Not specified	RR 42.9%	The entire cohort included 26 patients treated with cemiplimab (13 patients), pembrolizumab (7) and nivolumab (6)	In et al., 2020 [42]
57	46 M, 11 F; median age: 79–80 y	43 locoregional cSCCs; 14 m-cSCCs	200 mg IV every 3 weeks	4 CR (7%); 20 PR (35%); 10 SD (18%); 18 PD (32%)	CARSKIN trial (NCT02883556, phase II study)	Maubec et al., 2020 [65]
105	80 M, 15 F; median age: 72 y	47 recurrent cSCCs; 58 m-cSCCs	200 mg IV every 3 weeks	Overall RR 35.2%; 11 CR (10.5%); 26 PR (24.8%); 30 SD (28.6%); 28 PD (26.7%)	KEYNOTE-629 trial (phase II study), m-cSCC cohort	Grob et al., 2020 [62]; Hughes et al., 2021 [63]
54	39 M, 15 F; median age: 75.5 y	la-cSCCs	200 mg IV every 3 weeks	Overall RR 50%; 9 CR (16.7%); 18 PR (33.3%); 13 SD (24.1%); 9 PD (16.7%)	KEYNOTE-629 trial (phase II study), la-cSCC cohort	Hughes et al., 2021 [63]
20	17 M, 3 F; median age: 68 y	10 la-cSCCs; 10 m-cSCCs	200 mg IV every 3 weeks	Best overall RR 32%; 3 CR (16%); 3 PR (16%), 1 SD (5%); 12 PD (63%)	Subgroup analysis of trial NCT02721732	Ferrarotto et al., 2021 [66]
1	F, 62 y	m-BCC	Not specified	PD	/	Winkler et al., 2017 [59]
1	M, 77 y	Multiple BCCs	2 mg/kg IV every 3 weeks	PR	Patient with Gorlin-Goltz syndrome	Moreira et al., 2018 [74]
1	M, 81 y	m-BCC	2 mg/kg IV every 3 weeks	PR (near-complete response)	/	Fischer et al., 2018 [73]
1	M, in his 50 s	m-BCC	2 mg/kg IV every 3 weeks	CR	/	Cannon et al., 2018 [72]
16	Not specified	Advanced BCCs	Pembrolizumab monotherapy (2 mg/kg IV every 3 weeks) in 9 patients; pembrolizumab (2 mg/kg IV every 3 weeks) + vismodegib 150 mg orally daily in 7 patients.	RR: 44% (4/9 patients) for monotherapy group; RR: 29% (2/7 patients) for combination therapy group	/	Chang et al., 2019 [75]
1	Not specified	Advanced MCC	2 mg/kg IV every 3 weeks	CR	KEYNOTE-001 trial (NCT01295827, phase I study), involving 30 patients with advanced cancers treated with pembrolizumab	Patnaik et al., 2015 [76]
1	M, 80 y	m-MCC	2 mg/kg IV every 3 weeks	PR	/	Winkler et al., 2017 [78]
1	M, 59 y	m-MCC	2 mg/kg IV every 3 weeks	CR	/	Roche et al., 2017 [80]
1	M, 64 y	m-MCC	2 mg/kg IV every 3 weeks + RT	CR	/	Cugley et al., 2018 [77]
1	M, 62 y	m-MCC	2 mg/kg IV every 3 weeks	PR	Patient developed a pembrolizumab -associated mucous membrane pemphigoid and treatment was discontinued	Haug et al., 2018 [79]
2	M, 69 y M, 72 y	m-MCCs	Not specified + RT	2 PD	Patients had PD on pembrolizumab; CR and PR were achieved after palliative RT	Xu et al., 2018 [81]
1	M, 65 y	m-MCC	Not specified + RT	PD	Patient had PD on pembrolizumab; response was achieved after palliative RT; he developed cytokine release syndrome after RT	Barker et al., 2018 [82]
50	34 M, 16 F; median age: 70.5 y	7 la-MCCs; 43 m-MCCs	2 mg/kg IV every 3 weeks	Overall RR 58%; 15 CR (30%); 14 PR (28%); 4 SD (8%); 16 PD (32%)	Cancer Immunotherapy Trials Network-09/Keynote-017 phase II trial	Nghiem et al., 2021 [84]

Note: Abbreviations: M (male); F (female); y (years); la-cSCC (locally advanced cutaneous squamous cell carcinoma); m-cSCC (metastatic cutaneous squamous cell carcinoma); la-BCC (locally advanced basal cell carcinoma); m-BCC (metastatic basal cell carcinoma); la-MCC (locally advanced Merkel cell carcinoma); m-MCC (metastatic Merkel cell carcinoma); IV (intravenously); RT (radiotherapy); CR (complete response); PR (partial response); SD (stable disease); PD (progressive disease); RR (response rate).

**Table 4:** Reported cases of locally advanced and metastatic non-melanoma skin cancers treated with nivolumab.

Number of Patients	Patient Data (Sex, Age)	Diagnosis	Nivolumab Regimen	Outcome	Additional Information	Ref.
3	M, 65 y; M, 66 y; F, 61 y	2 m-cSCCs; 1 m-BCC (basosquamous cancer)	3 mg/kg IV every 2 weeks	1 PR; 2 SD	/	Borradori et al., 2016 [56]
1	F, age not specified	m-cSCC	3 mg/kg IV every 2 weeks	PR	/	Tran et al., 2017 [53]
1	F, 69 y	m-cSCC	Not specified	PR	Kidney transplant recipient; allograft preserved	Kittai et al., 2017 [95]
1	M, 74 y	la-cSCC	Nivolumab IV every 2 weeks + cetuximab every week	CR	/	Chen et al., 2017 [86]
3	M, 66 y; M, 72 y; F, 81 y	m-cSCCs	3 mg/kg IV every 2 weeks	3 PR (including one near-CR)	/	Blum et al., 2018 [85]
1	M, 50 y	m-cSCC	3 mg/kg IV every 2 weeks	PR	Kidney transplant recipient; allograft rejection	Goldman et al., 2018 [87]
6 (in a cohort of 26 patients)	Data for the entire cohort: 19 M, 7 F; median age: 64.5 y	Data for the entire cohort: 5 la-cSCCs; 21 m-cSCCs	Not specified	RR 33.3%	The entire cohort included 26 patients treated with cemiplimab (13), pembrolizumab (7) and nivolumab (6)	In et al., 2020 [42]
1	M, 58 y	m-BCC	240 mg IV every 2 weeks	PR (near-CR)	/	Ikeda et al., 2016 [88]
1	M, 42 y	m-MCC	3 mg/kg IV every 2 weeks	PR	/	Mantripragada et al., 2015 [89]
1	M, 80 y	m-MCC	3 mg/kg IV every 2 weeks	PR	/	Walocko et al., 2016 [90]
25		Advanced MCCs	240 mg IV every 2 weeks	3 CR (14%); 12 PR (55%); 4 SD (18%); 3 PD (14%)	CheckMate 358 study (phase I/II trial)	Topalian et al., 2017 [93]
1		m-MCC	3 mg/kg IV every month	PR	Kidney transplant recipient; allograft function preserved	Singh et al., 2019 [91]

Note: Abbreviations: M (male); F (female); y (years); m-BCC (metastatic basal cell carcinoma); la-cSCC (locally advanced cutaneous squamous cell carcinoma); m-cSCC (metastatic cutaneous squamous cell carcinoma); m-MCC (metastatic Merkel cell carcinoma); IV (intravenously); CR (complete response); PR (partial response); SD (stable disease); PD (progressive disease), RR (response rate).

**Table 5:** Reported cases of locally advanced and metastatic Merkel cell carcinomas treated with avelumab.

Number of Patients	Patient Data (Sex, Age)	Diagnosis	Avelumab Regimen	Outcome	Additional Information	Ref.
1	M, 85 y	m-MCC	Not specified	CR	/	Eshghi et al., 2018 [96]
1	M, 73 y	m-MCC	10 mg/kg IV every 2 weeks	PR	/	Zhao et al., 2018 [97]
88	65 M, 23 F; median age: 72.5 y	m-MCCs	10 mg/kg IV every 2 weeks	Objective RR 33%; 10 CR (11.4%); 19 PR (21.6%); 9 SD (10.2%); 32 PD (36.4%)	JAVELIN Merkel 200 trial (NCT02155647, phase II study, part A–previously treated m-MCC)	D’Angelo et al., 2020 [104]
116	81 M; 35 F; median age 74 y	m-MCCs	10 mg/kg IV every 2 weeks	Objective RR 39.7%; 19 CR (16.4%); 27 PR (23.3%); 12 SD (10.3%); 48 PD (41.4%)	JAVELIN Merkel 200 trial (NCT02155647, phase II study, part B–naïve m-MCC)	D’Angelo et al., 2021 [100]
102 (55 evaluable)	78 M, 23 F, 1 unknown; median age 70.6 y	m-MCCs	10 mg/kg IV every 2 weeks	55 evaluable patients; overall RR 29.1%; 6 CR (10.9%); 10 PR (18.2%); 17 SD (30.9%); 22 PD (40%)	Expanded access program compassionate use of avelumab	Grignani et al., 2021 [101]
367 (150 evaluable)	247 M, 119 F, 1 unknown; median age 71.6 y	m-MCCs	10 mg/kg IV every 2 weeks	150 evaluable patients; overall RR 48%; 38 CR (25.3%); 34 PR (22.7%); 37 SD (24.7%); 41 PD (27.3%)	Expanded access program compassionate use of avelumab	Ascierto et al., 2021 [102]
94 (28 patients receiving avelumab; 26 non-avelumab immunotherapy [19 pembrolizumab; 7 nivolumab], 40 chemotherapy)	64 M, 30 F; median age 73 y	27 la-MCCs; 67 m-MCCs	Not specified	Real-world overall RR 64.3% for avelumab; 61.5 for non-avelumab immunotherapy group	SPEAR-Merkel study	Bhanegaonkar et al., 2021 [105]
20	10 M, 10 F; median age:74 y	20 advanced MCCs	Not specified	Real-world overall RR 65%; 1 CR (5%); 13 PR (65%); 4 SD (20%); 2 PD (10%)	/	Badalamenti et al., 2022 [106]

Note: Abbreviations: M (male); F (female); y (years); m-MCC (metastatic Merkel cell carcinoma); IV (intravenously); CR (complete response); PR (partial response); SD (stable disease); PD (progressive disease); RR (response rate).

**Table 6:** Potential predictors to ICIs therapy in solid tumors and subdivided into different categories—starting from widely accessible to more specific ones.

Potential Predictors	Assessment	Difficult Access to Parameter Evaluation (Based on Patient Compliance, Cost, Availability)
Clinical features, including belonging to special populations (such as elderly, immunocompromised patients, organ transplant recipients, individuals suffering from autoimmune conditions)	Clinical	/
Serum markers	Blood-based	+
PD-L1 (and PD-1) expression	Histopathological, blood-based, molecular biology techniques	+/++
Tumor infiltrating lymphocytes	Histopathological, molecular biology techniques	+/++
Genetic alterations (tumor mutational burden and copy-number alterations)	Molecular biology techniques	++

**Table 7:** Possible predictors to immune checkpoint inhibitors response in locally advanced and metastatic cutaneous squamous cell carcinoma and basal cell carcinoma.

Potential Predictor and Its Assessment	Number of Patients and Type of Malignancy	Treatment	Value as Predictor	Ref.
Primary tumor location on the head/neck; clinical	26 advanced cSCCs (5 la-cSCC; 21 m-cSCC)	Cemiplimab (13 patients), pembrolizumab (7) and nivolumab (6)	Positive predictor (*p*-value = 0.04)	In et al., 2020 [42]
Primary tumor location on the leg; clinical	46 advanced cSCCs (25 la-cSCC; 21 m-cSCC)	Cemiplimab (8), pembrolizumab (28), nivolumab (10)	Negative predictor (*p*-value = 0.014)	Salzmann et al., 2020 [178]
Primary tumor location on the head/neck (A); performance status <2 (B); clinical	245 advanced cSCCs (85 la-cSCC; 159 m-cSCC, 1 unknown)	Cemiplimab	Positive predictors: ([A] *p*-value = 0.0001; [B] *p*-value = 0.0025)	Hober et al., 2021 [44]
Age > 60 years; clinical	18 advanced cSCCs (13 la-cSCC; 5 m-cSCC)	Cemiplimab	Positive predictor (*p*-value = 0.002)	Guillaume et al., 2021 [43]
Head/neck tumor location (A); normal haemoglobin values (B); clinical and blood-based	131 advanced cSCCs (91 la-cSCC; 40 m-cSCC)	Cemiplimab	Positive predictors: ([A] *p*-value = 0.007; [B] *p*-value = 0.034)	Baggi et al., 2021 [45]
Tumor location on the genitalia (A), treatment with any systemic antibiotic within 1 month of cemiplimab initiation (B), performance status ≥ 1 (C), chronic corticosteroids therapy (D), previous radiation therapy to lymph nodes (E); previous chemotherapy (F); clinical	131 advanced cSCCs (91 la-cSCC; 40 m-cSCC)	Cemiplimab	Negative predictors: ([A] *p*-value = 0.041; [B] *p*-value = 0.012; [C] *p*-value = 0.012; [D] *p*-value = 0.038; [E] *p*-value = 0.052; [F] *p*-value = 0.002)	Baggi et al., 2021 [45]
Head/neck tumor location (A); haemoglobin > 12 g/dL (B); clinical and blood-based	30 advanced cSCCs (25 la-cSCC; 5 m-cSCC)	Cemiplimab	Positive predictors: ([A] *p*-value = 0.016; [B] *p*-value = 0.042)	Strippoli et al., 2021 [51]
Absolute lymphocyte count; blood-based	61 advanced cSCCs (14 la-cSCCs; 47 m-cSCCs)	Cemiplimab, pembrolizumab, nivolumab	Positive predictor (*p*-value < 0.01)	Hanna et al., 2020 [179]
Elevated lactate dehydrogenase serum levels; blood-based	46 advanced cSCCs (25 la-cSCC; 21 m-cSCC)	Cemiplimab (8), pembrolizumab (28), nivolumab (10)	Negative predictor (*p*-value = 0.002)	Salzmann et al., 2020 [178]
Baseline low neuthophil/lymphocyte ratio + low platelet/lymphocyte ratio; blood-based	30 advanced cSCCs (25 la-cSCC; 5 m-cSCC)	Cemiplimab	Positive predictor	Strippoli et al., 2021 [51]
PD-L1 expression; immunohistochemical	48 la-cSCCs	Cemiplimab	Potential positive predictor	Migden et al., 2019 [41]
PD-L1 expression; immunohistochemical	16 advanced BCCs	Pembrolizumab monotherapy (9 patients), pembrolizumab + vismodegib (7)	Not confirmed as predictor	Chang et al., 2019 [75]
PD-L1 expression; immunohistochemical	57 advanced cSCCs (43 locoregional cSCCs and 14 m-cSCCs)	Pembrolizumab	Positive predictor (*p*-value = 0.02)	Maubec et al., 2020 [65]
PD-L1 expression; immunohistochemical	50 la-BCCs	Cemiplimab	Not confirmed as predictor	Stratigos et al., 2021 [12]
Low-level CNAs in the 3 q chromosomal arm; molecular biology techniques	33 advanced cSCC	Anti-PD-1, not specified	Positive predictor (*p*-value < 0.01)	Kacew et al., 2019 [119]
High TMB; molecular biology techniques	50 la-cSCCs	Cemiplimab	Potential positive predictor	Migden et al., 2019 [41]
High TMB; molecular biology techniques	61 advanced cSCCs (14 la-cSCCs; 47 m-cSCCs)	Cemiplimab, pembrolizumab, nivolumab	Positive predictor (*p*-value = 0.02)	Hanna et al., 2020 [179]
High TMB; molecular biology techniques	26 advanced cSCCs (5 la-cSCC; 21 m-cSCC)	Cemiplimab (13 patients), pembrolizumab (7) and nivolumab (6)	Positive predictor (*p*-value = 0.04)	In et al., 2020 [42]

Note: Abbreviations: TMB (tumor mutational burden); CNAs (copy-number alterations); la-BCC (locally advanced basal cell carcinoma; la-cSCC (locally advanced cutaneous squamous cell carcinoma); m-cSCC (metastatic cutaneous squamous cell carcinoma).

**Table 8:** Possible predictors to immune checkpoint inhibitor response in locally advanced and metastatic Merkel cell carcinoma.

Potential Predictor; Assessment	Number of Patients and Type of Malignancy	Treatment	Value as Predictor	Ref.
Previous lines of treatment; clinical	38 advanced MCCs	Pembrolizumab, nivolumab, avelumab, nivolumab + ipilimimab	Negative predictor (*p*-value = 0.0066)	Knepper et al., 2019 [17]
Unimpaired performance status, absence of immunosuppression; clinical	41 advanced MCCs	Pembrolizumab, nivolumab, avelumab	Positive predictors	Spassova et al., 2020 [19]
Impaired performance status (A), completion of 2 years of treatment(B); clinical	50 advanced MCCs	Pembrolizumab	A: negative predictor; B: positive predictor	Nghiem et al., 2021 [84]
Absence of immunosuppression, limited number of tumor-involved organ systems; clinical	114 unresectable MCCs	Pembrolizumab, nivolumab, avelumab	Negative predictors	Spassova et al., 2022 [194]
BMI ≥ 30; clinical	20 advanced MCCs	Avelumab	Positive predictor of longer time to treatment failure (*p*-value = 0.004) and objective RR (*p*-value = 0.01)	Badalamenti et al., 2022 [106]
MCPyV-specific B-cells and T-cells responses; blood-based	26 advanced MCCs	Pembrolizumab	Not confirmed as predictor, related to tumor burden	Miller et al., 2018 [195]
Reduction of neuron-specific enolase; blood based	23 advanced MCCs	Immunotherapy	Possible positive predictor	van Veenendaal et al. 2021 [196]
PD-L1 expression; immunohistochemical	88 m-MCCs	Avelumab	Not confirmed as predictor	Kaufman et al., 2018 [99]
PD-L1 expression; immunohistochemical	27 advanced MCCs	Pembrolizumab, nivolumab, avelumab, nivolumab + ipilimimab	Not confirmed as predictor (*p*-value = 0.606)	Knepper et al., 2019 [17]
PD-L1 expression; immunohistochemical	47 la- MCCs and m-MCCs	Pembrolizumab	Not confirmed as predictor (*p*-value = 0.68), associated to improved PFS and OS	Nghiem et al., 2019 [18]
PD-L1 expression; immunohistochemical	41 advanced MCCs	Pembrolizumab, nivolumab, avelumab,	Not confirmed as predictor	Spassova et al., 2020 [19]
PD-L1 expression; immunohistochemical	116 m-MCCs	Avelumab	Not confirmed as predictor, responses tended to be higher in PD-L1+ tumors	D’Angelo et al., 2021 [100]
PD-1+ cells density; immunohistochemical	26 advanced MCCs	Pembrolizumab	Positive predictor (*p*-value = 0.02)	Giraldo et al., 2018 [197]
PD-L1+ cells density; immunohistochemical	26 advanced MCCs	Pembrolizumab	Positive predictor (*p*-value = 0.03)	Giraldo et al., 2018 [197]
PD-1+/PD-L1+ cells proximity; immunohistochemical	26 advanced MCCs	Pembrolizumab	Positive predictor (*p*-value = 0.03)	Giraldo et al., 2018 [197]
PD-1 expression; immunohistochemical	27 advanced MCCs	Pembrolizumab, nivolumab, avelumab, nivolumab + ipilimimab	Positive predictor (*p*-value = 0.00598)	Knepper et al., 2019 [17]
TILs with low T-cell clonality and high TCR diversity; immunohistochemical and molecular biology techniques	41 advanced MCCs	Pembrolizumab, nivolumab, avelumab,	Positive predictor	Spassova et al., 2020 [19]
Increased intratumoral CD8+ T-cells; immunohistochemical	116 m-MCCs	Avelumab	Not confirmed as predictor, responses tended to be higher in tumors rich in CD8+ T-cells	D’Angelo et al., 2021 [100]
TILs rich in γδ T cells; flow cytometry from tumor suspension	39 advanced MCCs	Not specified, not used in all patients	Positive predictor in immunotherapy treated patients (*p*-value = 0.021)	Gherardin et al., 2021 [198]
Effector memory CD4+ and CD8+ T cells co-expressing CD28, HLA-DR and PD-1; blood-based immunophenotype	27 advanced MCCs	Pembrolizumab	Positive predictor (*p*-value < 0.05)	Greene et al., 2021 [199]
Predominance of CD8+ effector and central memory T-cells (A); T cells in proximity to tumoral cells (B); immunohistochemical	114 unresectable MCCs	Pembrolizumab, nivolumab, avelumab	Positive predictors ([A] *p*-value = 0.02; [B] *p*-value = 0.009)	Spassova et al., 2022 [194]
MCPyV positive status; immunohistochemical and molecular biology techniques	27 advanced MCCs	Pembrolizumab, nivolumab, avelumab, nivolumab + ipilimimab	Not confirmed as predictor (*p*-value = 0.63)	Knepper et al., 2019 [17]
MCPyV positive status; blood-based and immunohistochemical	47 la-MCCs and m-MCCs	Pembrolizumab	Not confirmed as predictor (*p*-value = 0.765)	Nghiem et al., 2019 [18]
MCPyV positive status; molecular biology techniques	41 advanced MCCs	Pembrolizumab, nivolumab, avelumab,	Not confirmed as predictor	Spassova et al., 2020 [19]
MCPyV positive status; molecular biology techniques	116 m-MCCs	Avelumab	Not confirmed as predictor, responses tended to be lower in MCPyV + tumors	D’Angelo et al., 2021 [100]

Note: Abbreviations: la-MCC (locally advanced Merkel cell carcinoma; m-MCC (metastatic Merkel squamous cell carcinoma); PFS (progression-free survival; OS (overall survival); TILs (tumor-infiltrating lymphocytes); TCR (T-cell receptor); MCPyV (Merkel cell polyomavirus).

## Data Availability

Data sharing not applicable.
